# Effects of aerobic exercise on body composition and exerkines in colorectal cancer survivors

**DOI:** 10.3389/fspor.2025.1579221

**Published:** 2025-06-26

**Authors:** Eunhan Cho, Miranda Chodzko, Stephanie L. E. Compton, Shengping Yang, Steven Heymsfield, Guillaume Spielmann, Justin C. Brown

**Affiliations:** ^1^ Department of Kinesiology, LSU School of Kinesiology, Baton Rouge, LA, United States; ^2^ Department of Population and Public Health Science, Pennington Biomedical Research Center, Baton Rouge, LA, United States; ^3^ Department of Interdisciplinary Oncology, LSU Health Sciences Center, New Orleans School of Medicine, New Orleans, LA, United States; ^4^ Stanley S. Scott Cancer Center, Louisiana State University Health Sciences Center, New Orleans, LA, United States; ^5^ Louisiana Cancer Research Center, New Orleans, LA, United States

**Keywords:** IL-7 biomarker, IL-15, exerkines, clinical trial, exercise intervention, body composition

## Abstract

**Introduction:**

Physical activity improves immune competency and is associated with a lower rate of cancer recurrence in colorectal cancer survivors. However, the exact mechanisms underlying these improvements remain unclear. Exercise-derived cytokines (exerkines), particularly IL-7 and IL-15, are crucial in maintaining optimal immune health. This study investigated whether a 12-week structured exercise training intervention increases IL-7 and IL-15 in colorectal cancer survivors.

**Methods:**

Sixty colorectal cancer survivors were randomized to a moderate-intensity home-based aerobic exercise group (150 min/week) or a control group for 12 weeks. IL-7 and IL-15 were quantified using ELISA, and body composition was measured using dual-energy x-ray absorptiometry.

**Results:**

At baseline, participants treated with chemotherapy had lower IL-7 than those not treated with chemotherapy [−3.3 pg/ml (95% CI: −1.3, −5.4); *p* = 0.002]. Baseline fitness capacity correlated with IL-15 (*r* = −0.37; *p* = 0.004). IL-7 increased in the exercise group [2.3 pg/ml (95% CI: 0.9, 3.8; *p* = 0.003)], but not in the control group [1.2 pg/ml (95% CI: 0.3, 2.8; *p* = 0.31)]. IL-15 did not differ between groups. Longitudinal changes in IL-15 were associated with changes in body composition.

**Discussion:**

Aerobic exercise may improve immune function in colorectal cancer survivors by restoring IL-7 after chemotherapy and improving IL-15 by altering body composition.

**Clinical Trial Registration:**

[https://clinicaltrials.gov/study/NCT03975491], identifier [NCT03975491].

## 1 Introduction

Participation in physical activity and maintaining a healthy body composition are associated with a lower risk of cancer recurrence and mortality in colorectal cancer survivors (1, 2). Physical activity includes any bodily movement produced by skeletal muscle that results in energy expenditure, whereas exercise is a subset of physical activity that is planned, structured, repetitive, and performed with the objective of improving or maintaining physical fitness (3). Recent evidence from a large, international, phase III randomized controlled trial demonstrated that exercise improves disease-free and overall survival in patients with colon cancer (4). However, the biological mechanisms that underpin how exercise and body composition causally relate to disease outcomes remain uncertain.

Exercise induces the release of soluble proteins—exerkines—from various tissues and organs that restore cellular communication and improve health (5). Interleukin (IL)-7 (IL-7) and IL-15 are particularly interesting exerkines because they maintain immune functions (6, 7). IL-7 is essential for the development, maintenance, and survival of T-cells (8), while IL-15 contributes to the development and maintenance of memory CD8+ T-cells and modulates the function of Natural Killer (NK) cells (9, 10). IL-7 is primarily produced by skeletal muscle, whereas IL-15 is acutely secreted by skeletal muscle during contraction and chronically secreted by adipose tissue (11).

To clarify the mechanistic relevance of IL-7 and IL-15 in colorectal cancer, we conducted a secondary analysis of the Exercise and Colorectal Cancer Treatment (EXACT) trial. We hypothesized that 12 weeks of moderate-intensity aerobic exercise compared with a control condition would restore circulating IL-7 and IL-15 in colorectal cancer survivors in a pattern compatible with an improved cancer prognosis.

## 2 Materials and methods

### 2.1 Study design

**Figure 1:**
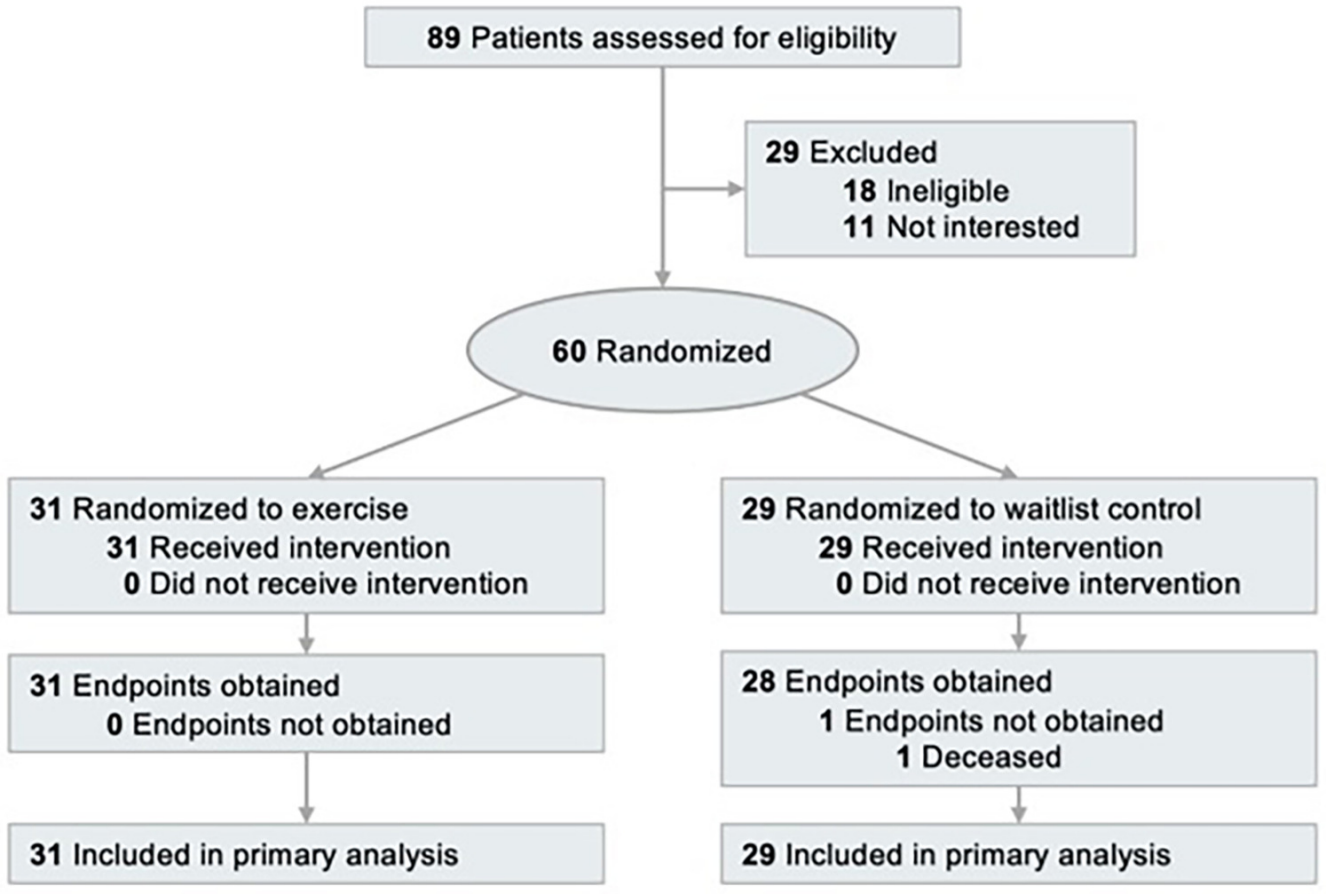
Flow chart of the clinical trial [(http://clinicaltrials.gov/) identifier: NCT03975491].

The study used a randomized, parallel-group, controlled design conducted at a single site (Pennington Biomedical Research Center, Baton Rouge, LA). The Institutional Review Board approved the protocol and informed consent document (PBRC-2019-009). All participants provided written informed consent. The study was registered on ClinicalTrials.gov as NCT03975491. The trial design is described elsewhere (12). The flowchart of participant selection is presented (Figure 1).

#### 2.1.1 Participants

Potentially eligible participants were identified through the Louisiana Tumor Registry and contacted via mail. Inclusion criteria required participants to be 18 years or older with histologically confirmed stage I-III colorectal cancer, having completed surgical resection, if applicable, completed chemotherapy or radiotherapy more than one month before study enrollment, and self-reported less than 90 min of moderate to vigorous physical activity per week over the past month.

#### 2.1.2 Randomization and blinding

Participants were stratified by sex and randomized in a 1:1 ratio to an exercise group or a wait-list control group using a computer-generated algorithm implemented by the study biostatistician. Outcome assessors were blinded to treatment. Participants and the certified cancer exercise specialists were not blinded.

#### 2.1.3 Interventions

The exercise group completed a 12-week moderate-intensity (50%–70% of age-predicted maximum heart rate) aerobic exercise program. Each participant received an in-home treadmill (T202, Horizon Fitness, Cottage Grove, WI, USA) and a heart rate monitor (Polar M430) (12). A certified cancer exercise specialist contacted participants to explain the exercise prescription, which included a 5 min warmup of slow walking, 30–60 min of moderate-intensity walking, and a 5 min cooldown of slow walking. The prescribed weekly exercise volume was incrementally increased by 30 min over the first four weeks until participants completed 150 min of exercise per week (12). The exercise specialist downloaded the heart rate monitor information weekly to review the frequency, duration, and intensity of exercise to ensure adherence to the protocol. Those not meeting the exercise study expectations were reminded of their goals and could discuss any barriers with the cancer exercise specialist. Exercise adherence was calculated as the total number of minutes of moderate-intensity exercise completed divided by the total number of minutes prescribed over the 12-week intervention period; this value was capped at 100% for each participant (12).

The waitlist control group was instructed to maintain their usual physical activity levels. A certified cancer exercise specialist contacted participants weekly to ensure ongoing communication about the study and monitor for adverse events. The exercise specialist did not provide exercise guidance during this period. After completing 12-week assessments, control group participants received an in-home treadmill and a personalized exercise program like that of the exercise group.

#### 2.1.4 Exerkine measurements

Resting blood was collected after a 10 h fast. Post-intervention blood draws occurred 24–72 h after the last exercise session. Blood samples were centrifuged and stored at −80 °C until batch analyses were performed at the end of the study. Concentrations of IL-7 (ab270218; Abcam, MA, USA) and IL-15 (#41702; PBL Assay Science, NJ, USA) were quantified using Enzyme-Linked Immunosorbent Assays (ELISA). Plasma samples and ELISA kit components were equilibrated to room temperature before analysis. Sample dilutions were performed using the assay buffer according to the specific instructions provided in each ELISA kit. Wash buffer and standard solutions were freshly prepared, following the manufacturer's guidelines. All samples and standards were analyzed in duplicate. The coefficient of variation (CV) for duplicate samples was maintained below 10%. The lower limits of detection were 5.9 pg/ml for IL-7 and 0.51 pg/ml for IL-15.

#### 2.1.5 Body composition and anthropometric measurements

Height (meters) was measured using a stadiometer, and weight (kilograms) was measured using calibrated digital scales with a participant wearing a medical gown. Body mass index (BMI) was calculated as weight (kg) divided by height squared (m^2^). Waist circumference was measured at the midpoint between the lower margin of the last palpable rib and the top of the iliac crest using a flexible, non-stretchable tape measure. The measurements were taken with the subject standing upright, feet together, and arms at the sides. Whole-body dual-energy x-ray absorptiometry (DXA) was performed to quantify bone mineral content, lean soft tissue mass, and fat mass using the Horizon A system equipped with Apex software v5.5 (Hologic, Bedford, MA, USA).

#### 2.1.6 Cardiorespiratory fitness assessment

Cardiorespiratory fitness was assessed using a modified Bruce Protocol on an electronic motorized treadmill (13). The test continued until participants reached 80% of their age-predicted maximum heart rate or reported a rating of perceived exertion of 18, per the American College of Sports Medicine's Guidelines for Exercise Testing and Prescription (14). Throughout the test, heart rate, blood pressure, and ratings of perceived exertion were monitored. The workload, expressed in metabolic equivalents (METs), at 80% of the age-predicted maximum heart rate (or RPE 18), was used to measure submaximal exercise capacity.

### 2.2 Statistical analysis

All statistical analyses were performed using JMPro 18 (SAS Inc., Cary, NC), and graphical representations were constructed using GraphPad Prism 10.00 (GraphPad Software). Biomarker concentrations are presented as mean ±95% confidence intervals. Differences in outcome measures (IL-7 and IL-15) between groups (exercise vs. control) over time (baseline vs. week 12) were tested using two-way repeated-measures analysis of variance (RM-ANOVA), adjusted for sex (randomized stratification factor) and baseline value of the dependent variable. Pearson's correlations were used to evaluate the relationship between IL-7 or IL-15 and body composition at baseline. Additional analysis examined the relationships between changes in fitness capacity, body composition, and IL-7 and IL-15 across all participants. Data normality was assessed through both visual inspection of Q–Q plots and histograms, as well as formal testing using the Shapiro–Wilk test. Statistical significance was set at 0.05. As IL-7 and IL-15 were exploratory outcomes, no multiple testing adjustment was applied, and the results should be considered hypothesis-generating.

## 3 Results

### 3.1 Baseline characteristics

#### 3.1.1 Participant characteristics

Between August 2019 and February 2023, 60 participants were randomized. Baseline characteristics were balanced between groups; participants had a mean age of 60.5 ± 10.9 years, 65.0% were female, with 26.7%, 28.3%, and 45.0% with stage I, II, and III cancer, respectively (Table 1). At week 12, biospecimens were available for analysis from 59 participants. The average adherence to the exercise prescription was 92.2% (12).

**Table 1:** Participant demographic, clinical, and physical activity characteristics at baseline.

Characteristic	Control (*n* = 29)	Exercise (*n* = 31)
Age, years	58.6 ± 10.6	62.3 ± 10.9
Sex, *n* %		
Male	10 (35%)	11 (35%)
Female	19 (65%)	20 (65%)
Race, *n* (%)		
White	23 (79%)	22 (71%)
Black	4 (14%)	6 (19%)
Other	2 (7%)	3 (10%)
Primary tumor, *n* (%)		
Colon	22 (76%)	24 (77%)
Rectal	7 (25%)	7 (23%)
Cancer treatment, *n* (%)		
Chemotherapy	14 (48%)	19 (61%)
Radiotherapy	4 (14%)	6 (19%)
Cancer stage, *n* (%)		
I/II	17 (59%)	16 (52%)
III	12 (41%)	15 (48%)
Accelerometry, min/d		
Sedentary, mean (SD)	543.2 ± 77.0	557.9 ± 153.3
Light intensity, mean (SD)	215.8 ± 69.0	190.6 ± 52.7
Moderate intensity, mean (SD)	25.6 ± 32.7	21.4 ± 19.9
Vigorous intensity, mean (SD)	0.2 ± 0.3	0.3 ± 1.2

Data are mean (SD) or *n* (%).

#### 3.1.2 Baseline factors associated with IL-7

**Figure 2:**
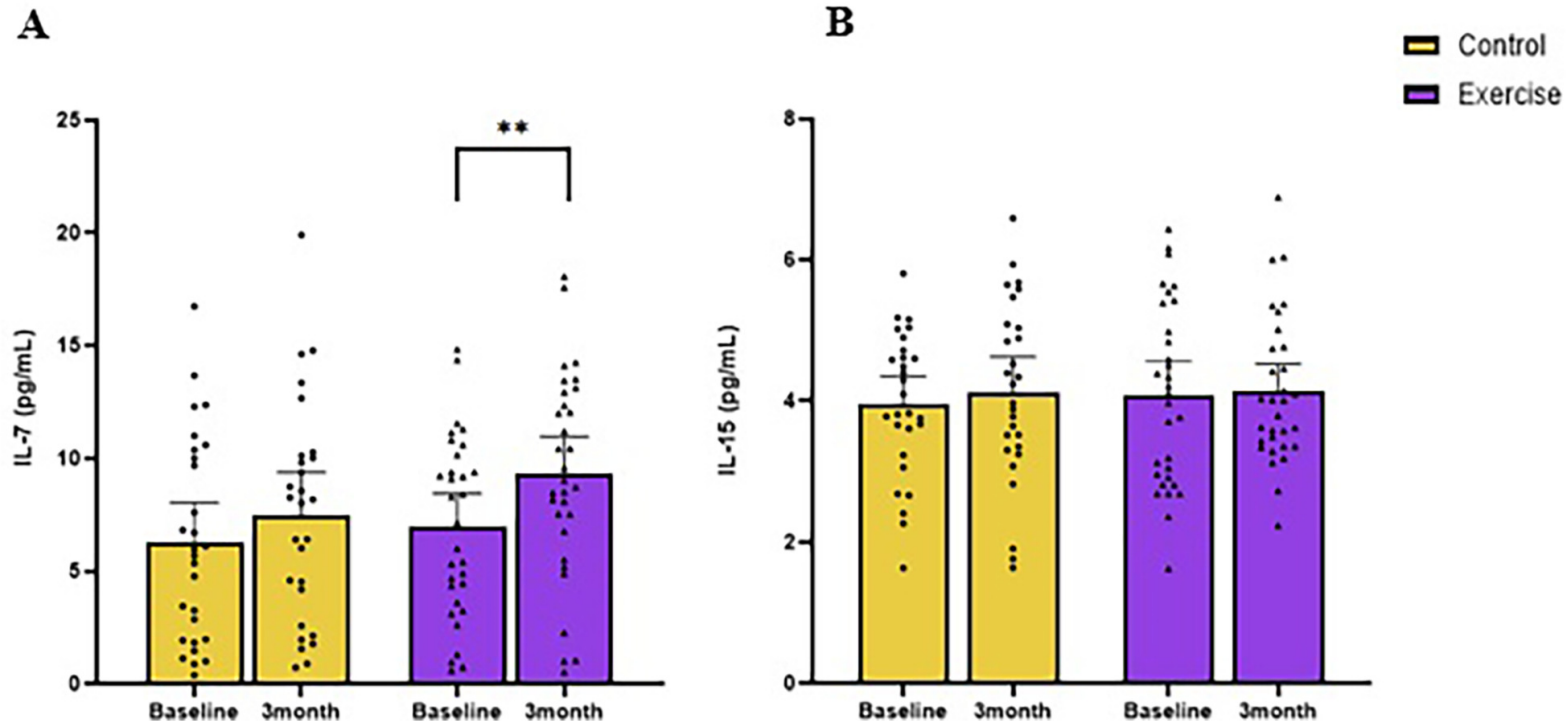
Changes in resting circulating IL-7 **(A)** and IL-15 **(B)** following 3 months of moderate-intensity aerobic exercise (purple) or wait-list control (yellow) in colorectal cancer survivors. **indicate values of *p* < 0.01 different from baseline.

**Figure 3:**
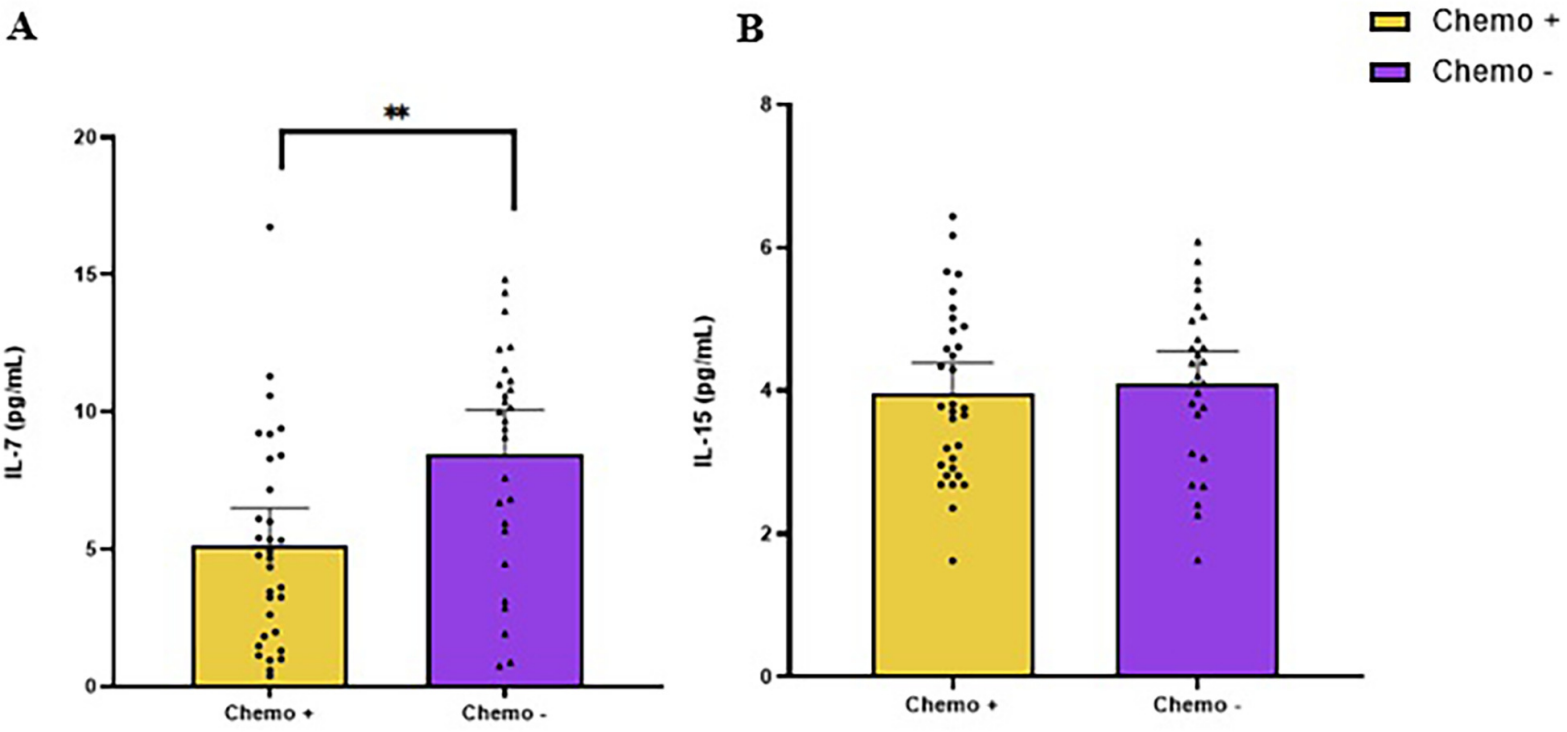
Baseline levels of IL-7 **(A)** and IL-15 **(B)** in colorectal cancer survivors, comparing participants who underwent chemotherapy (chemo+) with those who did not (chemo−). ** indicate values of *p* < 0.01 different from chemo−.

The concentration of IL-7 at baseline for exercise and control was 6.5 pg/ml (95% CI: 5.4, 7.6) and 6.6 pg/ml (95% CI: 5.6, 7.6), respectively (Figure 2A). Patients treated with chemotherapy had lower IL-7 than those not treated with chemotherapy [−3.3 pg/ml (95% CI: −1.3, −5.4); *p* = 0.002; Figure 3].

#### 3.1.3 Baseline factors associated with IL-15

**Figure 4:**
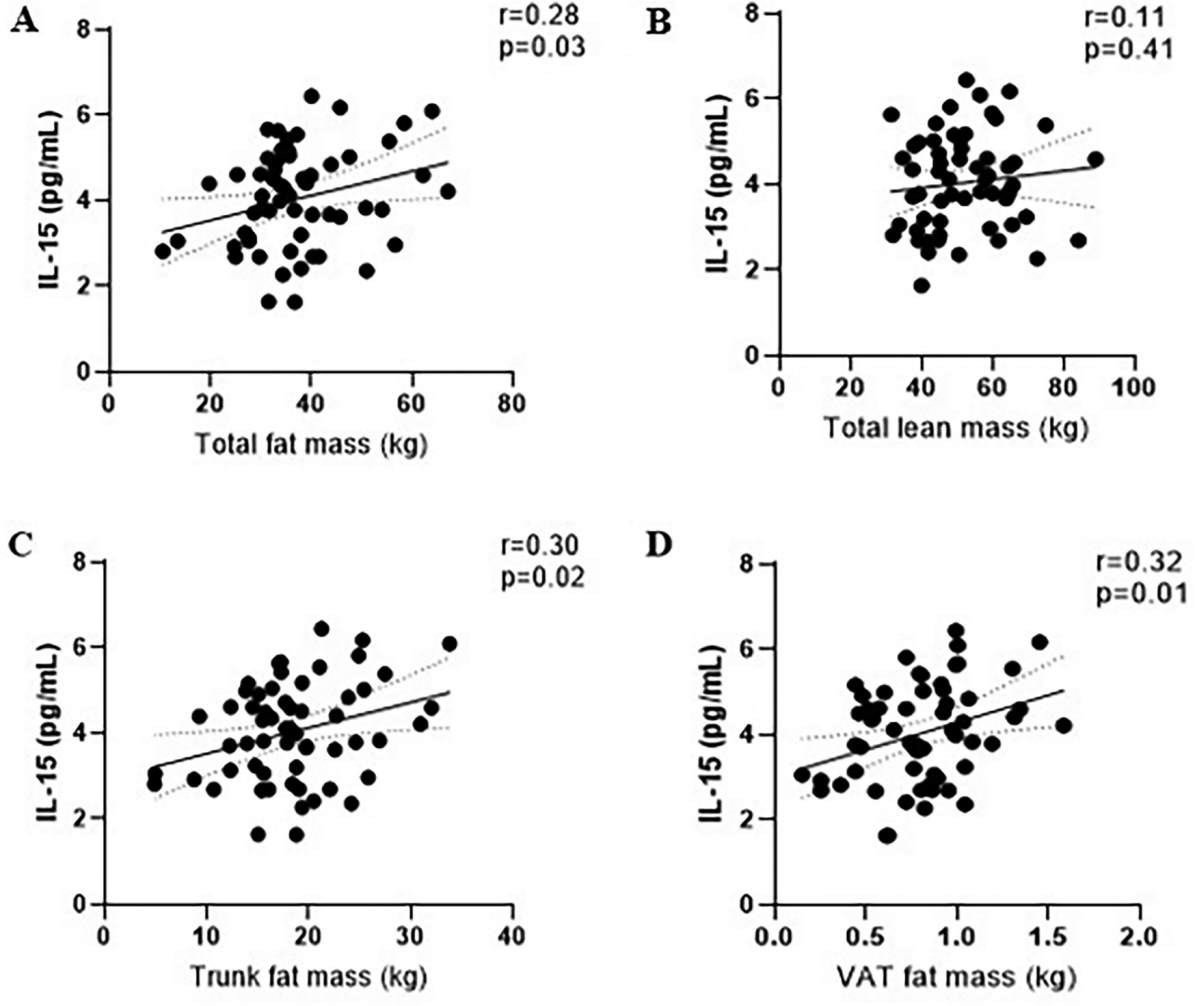
Correlations between baseline of IL-15 level and fat mass **(A)**, lean mass **(B)**, trunk fat mass **(C)**, and visceral adipose tissue mass **(D)**.

The concentration of IL-15 at baseline for exercise and control were 4.0 pg/ml (95% CI: 3.9, 4.2) and 4.0 pg/ml (95% CI: 3.9, 4.2), respectively (Figure 2B). Baseline aerobic fitness was inversely associated with IL-15 (*r* = −0.37, *p* = 0.004). Baseline BMI (*r* = 0.31, *p* = 0.02), waist circumference (*r* = 0.29, *p* = 0.02), total body fat mass (*r* = 0.28, *p* = 0.03), trunk fat mass (*r* = 0.30, *p* = 0.02), trunk fat percentage (*r* = 0.26, *p* = 0.04), android fat mass(*r* = 0.33, *p* = 0.01), and volume of visceral adipose tissue (VAT) (*r* = 0.32, *p* = 0.01) were positively correlated with baseline IL-15 (Figure 4).

### 3.2 Exerkines and body composition following 12 weeks of aerobic exercise

#### 3.2.1 Changes in body composition in response to exercise training

After 12 weeks, there were no statistically significant differences between the exercise and control groups in weight [0.86 kg (95% CI:−0.62, 2.33); *p* = 0.25; (Table 2)] or BMI [0.38 kg/m^2^ (95% CI −0.14, 0.9); *p* = 0.15]. Additionally, there were no statistically significant differences in DXA outcomes.

**Table 2:** Anthropometric and body composition measurements.

Measurements	Group	Baseline (mean ± SD)	Δ Baseline to week 12 (mean ± 95% CI)	Δ from control (mean ± 95% CI)	*p*-value
Weight, kg	Control	90.6 ± 13.0	−1.21 (−2.28, −0.15)	0.00—Referent	—
	Exercise	91.6 ± 25.6	−0.35 (−1.38, 0.67)	0.86 (−0.62, 2.33)	0.25
Body Mass Index, kg/m^2^	Control	32.1 ± 4.1	−0.49 (−0.87, −0.11)	0.00—Referent	—
	Exercise	32.9 ± 7.5	−0.11 (−0.47, 0.25)	0.38 (−0.14, 0.9)	0.15
Waist Circumference, cm	Control	103.8 ± 11.0	−3.60 (−6.62, 0.58)	0.00—Referent	—
	Exercise	105.4 ± 19.1	−1.02 (−3.91, 1.87)	2.58 (−1.60, 6.76)	0.22
Trunk Fat Mass, kg	Control	18.2 ± 4.0	−0.53 (−0.93, −0.14)	0.00—Referent	—
	Exercise	18.8 ± 7.2	−0.24 (−0.62, 0.14)	0.29 (−0.26, 0.84)	0.29
Android Fat Mass, kg	Control	3.3 ± 8.3	−0.13 (−0.22, 0.03)	0.00—Referent	—
	Exercise	3.6 ± 1.6	−0.07 (−0.16, 0.03)	0.06 (−0.08,0.20)	0.40
Total Fat Mass, kg	Control	36.3 ± 8.3	−0.79 (−1.49, 0.09)	0.00—Referent	—
	Exercise	37.9 ± 13.5	−0.40 (−1.07, 0.28)	0.39 (−0.62,1.40)	0.44
Total Fat, %	Control	40.2 ± 6.9	−0.25 (−0.71, 0.20)	0.00—Referent	—
	Exercise	40.9 ± 7.5	−0.16 (−0.60, 0.28)	0.09 (−0.56, 0.75)	0.77
VAT Fat Mass, kg	Control	0.8 ± 0.2	−0.00 (−0.08, 0.00)	0.00—Referent	—
	Exercise	0.8 ± 0.3	−0.00 (−0.05, 0.04)	0.04 (−0.02, 0.09)	0.23
SAT Fat Mass, kg	Control	2.2 ± 0.6	−0.06 (−0.11, 0.00)	0.00—Referent	—
	Exercise	2.2 ± 0.8	−0.03 (−0.09, 0.02)	0.02 (−0.06, 0.11)	0.56
Total Lean Mass, kg	Control	51.8 ± 10.3	−0.59 (−1.18, 0.00)	0.00—Referent	—
	Exercise	51.1 ± 14.7	−0.02 (−0.58, 0.55)	0.571 (−0.25, 1.39)	0.17
Bone Mineral Density, g/cm^3^	Control	1.1 ± 0.1	0.01 (−0.00, 0.02)	0.00—Referent	—
	Exercise	1.1 ± 0.1	0.00 (−0.01, 0.01)	−0.01 (−0.02, 0.00)	0.11

Data presented as Mean (SD).

VAT, visceral adipose tissue; SAT, subcutaneous adipose tissue; SD, standard deviation; CI, confidence interval.

#### 3.2.2 Changes in resting IL-7 in response to exercise training

After 12 weeks, IL-7 in the exercise group increased from baseline [2.3 pg/ml (95% CI: 0.9, 3.8; *p* = 0.003; Figure 2A), whereas the control group had no change [1.2 pg/ml (95% CI: 0.3, 2.8; *p* = 0.313)]. The longitudinal between-group difference (Δ) in IL-7 did not differ [0.7 pg/ml (95% CI: −1.5, 2.9); *p* = 0.53].

#### 3.2.3 Changes in resting IL-15 in response to exercise training

After 12 weeks, IL-15 did not change in the exercise group [−0.1 pg/ml (95% CI: −0.3, 0.2); *p* = 0.62] or the control group [−0.2 pg/ml (95% CI: −0.4, 0.1); *p* = 0.15]; Figure 2B].

### 3.3 Change in fitness and body composition moderates the changes in resting IL-15

Changes in aerobic fitness and IL-15 were correlated with changes in various body composition measures, including trunk fat percentage (*p* = 0.009), android fat percentage (*p* = 0.02), gynoid fat mass (*p* = 0.02), total fat percentage (*p* = 0.006), and subtotal fat percentage (*p* = 0.005). Changes in subcutaneous adipose tissue (SAT) volume, mass, and area (all *p* = 0.03) also showed moderation effects in the relationship between the change in aerobic fitness with the change in IL-15.

## 4 Discussion

This study found that patients previously treated with chemotherapy had lower circulating IL-7 concentrations at baseline compared to those who had not received chemotherapy. The 12-week exercise intervention increased circulating IL-7 concentrations in colorectal cancer survivors, irrespective of chemotherapy history. Additionally, while the 12-week, 150-minute/week aerobic intervention did not affect circulating IL-15 levels, we observed a correlation between changes in IL-15 with alterations in body fat mass, particularly with visceral adipose tissue (VAT).

Radiotherapy and chemotherapy are potent treatments designed to kill cancer cells but inadvertently damage healthy cells locally and systemically (15). Compared to those who have not undergone chemotherapy, participants who received chemotherapy may selectively impair IL-7-mediated immune pathways, such as T-cell homeostasis and survival, without broadly suppressing all immune functions (16, 17). However, regular aerobic exercise may help restore immune-related processes that are important for T-cell development and homeostasis (8). Given that exogenous IL-7 has been used as immunotherapy in cancer clinical trials to reconstitute the weakened immune system following aggressive treatments (18), our findings suggest that aerobic exercise could be an effective intervention to restore IL-7.

Our study showed no between-group difference in IL-15 following the 12-week intervention. This finding is consistent with other studies in cancer survivors, including breast, prostate, and colorectal cancer survivors (19). Interestingly, we observed correlations between baseline IL-15 with body composition parameters, specifically in the abdomen. Notably, these correlations were not found with subcutaneous adipose tissue (SAT), consistent with another study involving healthy adults over 70 years that reported similar correlations between IL-15 and total abdominal fat and VAT as measured by CT scan (20). RNA levels of IL-15 and IL-15 receptor alpha (IL15Rα) are known to be higher in SAT and VAT than in skeletal muscle (20), while the level of IL-15 secretion can vary depending on the timing of the measurement after exercise cessation (21, 22). Several studies have suggested that individuals with obesity have higher IL-15 compared to lean individuals (20, 23). Similarly, patients with coronary artery disease had a higher IL-15 and IL-15α, along with increased abdominal fat accumulation, compared to patients without coronary artery disease, suggesting that adipose tissue rather than muscle tissue, may be the primary source of IL-15 production (23).

Based on our findings of an inverse association between aerobic fitness and IL-15, coupled with the observed increase in aerobic fitness following the 12-week intervention, we conducted a moderation analysis to investigate whether improved METs influenced changes in body composition. Our moderation analysis revealed significant interactions between changes in METs, IL-15, and various body composition parameters. Specifically, we observed that the relationship between change in aerobic fitness and IL-15 was moderated by alterations in trunk fat percentage, changes in android fat percentage, and changes in total fat percentage. The moderation effect indicates that the impact of improved exercise capacity on IL-15 may vary depending on concurrent changes in body composition, particularly in central adiposity.

While IL-15 is primarily released from skeletal muscle tissue (i.e., myokines) during exercise (5), the mechanisms governing its secretion into the bloodstream remain unclear. Studies have shown that increased gene expression of IL-15 does not necessarily translate to corresponding increases in IL-15 protein levels, suggesting that an intricate modulation or mediators regulate circulating IL-15 (24, 25). The complexity of this relationship is further highlighted by a meta-analysis of 18 studies investigating the long-term effects of exercise on circulating IL-15 [standardized mean difference = 0.4 (95% CI: −0.08 to 0.88), *p* = 0.10] (26). However, a few studies have shown increased IL-15 following exercise training. For example, a study involving 116 patients with stage I–III breast and colorectal cancer, randomized to 12 weeks of either aerobic exercise or metformin pharmacotherapy, demonstrated dichotomic effects on IL-15. In this study, participants assigned to exercise showed increases in IL-15 compared to non-exercisers, while those assigned to metformin exhibited decreased IL-15 compared to their non-metformin-treated counterparts (27). However, without body composition measurements in this study, we cannot identify how these interventions influenced body fat and skeletal muscle in relation to IL-15. A cross-sectional study showed that the physically active elderly group had higher concentrations of circulating resting IL-15 than sedentary participants, which was associated with a higher ratio of naïve/memory T-cells, indicating that physically fit individuals tend to preserve “younger” immune T-cell phenotypes and functions (28). The discrepancies observed between cross-sectional and longitudinal studies suggest that lifestyle factors or excessive body fat may affect cytokine release throughout the body and play complex roles in modulating IL-15. The relationship between IL-15, inflammation, and adiposity appears to be multifaceted, given the different sources, and timing of IL-15 production from skeletal muscle and adipose tissue. While skeletal muscle secretes IL-15 immediately after exercise for up to 60 min, adipose tissue can constitutively release IL-15, thus contributing more to systemic IL-15 levels than skeletal muscle tissue (20).

This study has several limitations. The relatively short 12-week duration of the intervention may have restricted the observation of long-term changes in IL-15 levels and body composition, potentially not capturing the full extent of exercise-induced adaptations on exerkines. The lack of acute IL-15 measures immediately following exercise limited our understanding of IL-15 secretion dynamics in response to an acute exercise bout. Furthermore, the absence of direct measurements of muscle mass and strength hindered the establishment of a clear relationship between skeletal muscle adaptations and IL-15. Importantly, we did not adjust for multiple comparisons. Therefore, the findings should be considered hypothesis-generating.

Despite these limitations, the approach to investigating both IL-7 and IL-15 provides valuable insights into the effects of exercise on immune-related exerkines in colorectal cancer survivors. By examining these key exerkines, the current study offers a clearer picture of how exercise influences immune function in this population. The inclusion of participants with diverse cancer stages and treatment histories enhances the study's external validity, making the findings more generalizable to a broader range of colorectal cancer survivors.

In conclusion, 12-week aerobic exercise was associated with increased circulating IL-7 concentrations in colorectal cancer survivors. Patients who had previously received chemotherapy had lower IL-7 at baseline, and exercise intervention increased IL-7. While our findings on IL-7 are clear, the role of IL-15 as a beneficial “exerkine” requires further investigation. Future studies should adopt a more comprehensive approach by assessing IL-15 changes before and after acute bouts of exercise, and how the acute response to exercise may change after chronic exercise training.

## Data Availability

The original contributions presented in the study are included in the article/Supplementary Material, further inquiries can be directed to the corresponding author.
